# Emphasis on the Optimal Functional Recovery Through a Structured Inpatient Rehabilitation Program Along With a Home Exercise Regime in an Individual With Miller-Fisher Syndrome: A Case Report

**DOI:** 10.7759/cureus.29919

**Published:** 2022-10-04

**Authors:** Manali A Boob, Ragini Dadgal, Vikrant G Salphale

**Affiliations:** 1 Physiotherapy, Ravi Nair Physiotherapy College, Datta Meghe Institute of Medical Sciences, Wardha, IND; 2 Neurophysiotherapy, Ravi Nair Physiotherapy College, Datta Meghe Institute of Medical Sciences, Wardha, IND

**Keywords:** frenkel's, guillain-barre syndrome, physical rehabilitation, hughes severity scale, miller-fisher syndrome

## Abstract

Guillain-Barre syndrome (GBS) is a rare inflammatory demyelinating polyradiculoneuropathy characterized by motor impairment, progressive, ascending, symmetrical flaccid limb paralysis, areflexia or hyporeflexia, and with or without cranial nerve involvement, which are the hallmark clinical indications of GBS, which can last over weeks to months. Miller-Fisher syndrome (MFS) is a post-infectious localized variant of GBS that includes ophthalmoplegia, ataxia, and areflexia, and is often associated with lower cranial and facial nerve involvement. In this case, a 22-year-old young man was taken to a hospital after 10 days with complaints of bilateral symmetrical upper extremity and lower extremity paralysis, with the legs being more afflicted than the arms. For the past six days, he had an episode of fever, slurred speech, bilateral eye drops, and swallowing difficulty. On examination, the patient was identified with MFS, a variant of GBS. On the first and last day of treatment, the patient's outcome measures were recorded on Manual Muscle Testing, Hughes (GBS disability score), and the Functional Independence Measure Scale. Treatment options have been shown to reduce challenges and improve patient outcomes and quality of life, all of which are important at this stage. This case study concluded with a rehabilitation program that helped the patient to enhance his strength, range of motion, functional mobility, postural control, balancing abilities, weight-bearing, and prevent secondary impairments.

## Introduction

Guillain-Barre syndrome (GBS) is an uncommon inflammatory, demyelinating polyradiculoneuropathy that causes motor weakness. Seventy to eighty percent of cases are the most prevalent type [1]. The variants of GB syndrome include acute inflammatory demyelinating neuropathy (AIDP), acute motor axonal neuropathy (AMAN), acute motor and sensory axonal neuropathy (AMSAN), Miller-Fisher syndrome (MFS), polyneuritis cranialis (PNC), the pharyngeal cervical and brachial (PCB) variant, and Bickerstaff brainstem encephalitis (BBE) [2]. The hallmark clinical signs of GBS include progressive, ascending, symmetrical flaccid limb paralysis, areflexia, or hyporeflexia, with or without cranial nerve involvement, which can occur over weeks to several months [2]. Two-thirds of GBS individuals frequently report a pulmonary or gastric illness 2-4 weeks before the beginning of neurological issues [3]. The inclusion of the diaphragm leads to respiratory failure and, eventually, death [3]. GBS is now the most prevalent cause of acute flaccid paralysis worldwide, with a baseline incidence of 1.11 per 100,000 persons per year, with poliomyelitis under control [4]. MFS is a post-infectious localized type of GBS that includes ophthalmoplegia, ataxia, and areflexia and is commonly associated with the involvement of the facial and lower cranial nerves [5]. Bickerstaff brainstem encephalitis causes alternate consciousness, ataxia, ophthalmoplegia, and paradoxical hyperreflexia [6]. Antibodies to ganglioside Q1b (GQ1b) have been linked to Fisher's syndrome. Antibodies to GQ1b have been shown to damage motor nerve terminals in vitro via a complement-mediated mechanism [7]. This case report suggested a 22-year-old man complained of paralysis in all four limbs, slurred speech, drooping eyelids, and inability to conduct coordinated movement, as well as a history of fever, and was diagnosed with MFS, a variant of GBS. Physical therapy can help with this illness by lowering symptoms and improving functional outcomes and quality of life, which are all necessary for the long term.

## Case presentation

Here, we reported a case of a 22-year-old man who was admitted to a hospital with a complaint of bilateral symmetrical upper and lower extremity weakness, with the legs being more affected than the arms, over the previous 10 days. He had experienced fever, slurred speech, bilateral eye drops, and swallowing difficulties for the last six days. For the past three days, the patient had difficulty in walking and micturition and was bedridden with a catheter in place. The patient had no history of any infection, head injuries, hemoptysis, seizures, or ENT bleeding. The patient had no relevant history of diabetes, hypertension, bronchial asthma, or tuberculosis. The patient had no relevant family history. The evaluation and investigation verified that it was a case of MFS, which is a variant of GBS. As a result, the patient was hospitalized in the neuro ICU at Acharya Vinoba Bhave Rural Hospital (AVBRH), Wardha, India, for specialized treatment and referred to neuro physiotherapy for further care.

Clinical examination

**Figure 1:**
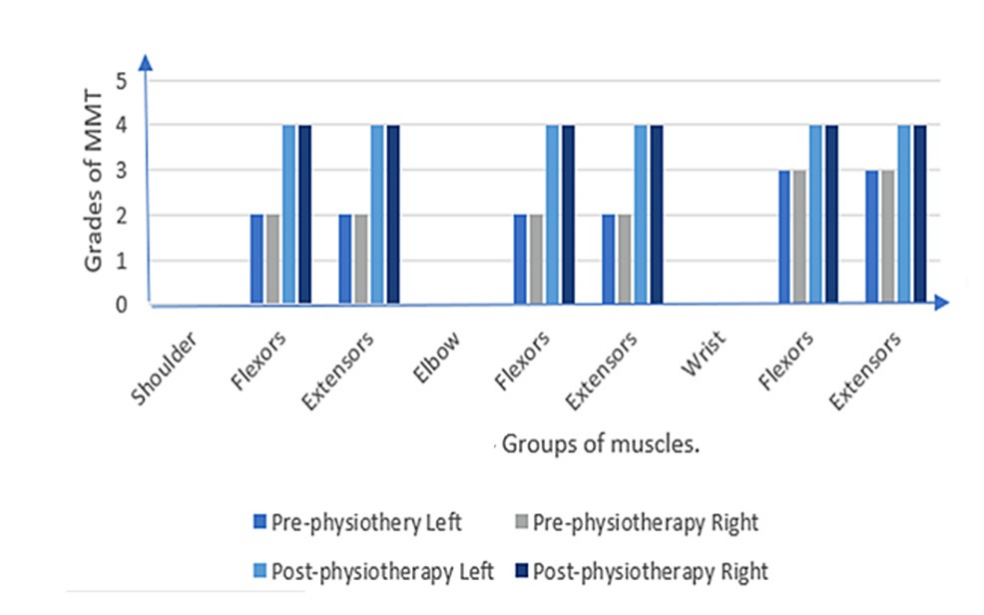
A graphical representation of manual muscle testing of upper limb

**Figure 2:**
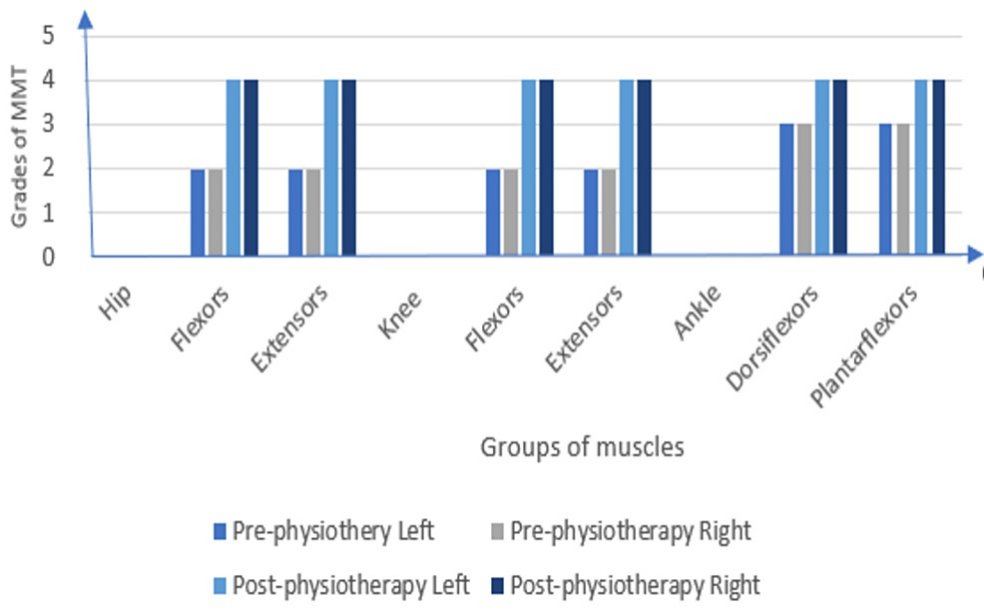
A graphical illustration of lower-limb manual muscle testing

The patient's oral consent was taken before beginning the examination procedure. On inspection and observation, the following were noted: The patient was observed in the supine lying position. The patient was ectomorph in his body type and was awake, following instructions. As the patient had difficulty in breathing, he was dependent on his accessory muscles for respiration. Ptosis was seen on both sides. The patient was reliant on Ryles tube for his feeding because biting, chewing, swallowing, and tongue movements were difficult due to injuries to the trigeminal, glossopharyngeal, and hypoglossal nerves. The III, IV, VI, and VII cranial nerves were impaired in their function. To facilitate the drainage of urine, a Foley catheter was inserted. Palpation corroborated all of the findings of the inspection, and the vitals were within normal limits. The ocular muscles were paralyzed, indicating ophthalmoplegia. Superficial and deep sensations were intact. The upper and lower limbs had a normal tone. Deep reflexes were absent, whereas superficial reflexes were diminished (Table 1). The patient had impaired coordinated movement and reduced strength when assessed on Manual Muscle Testing (MMT) as shown in Figure 1 and Figure 2; on the X-axis, groups of muscles are noted and on the Y-axis, grades of MMT are noted. The patient was unable to execute the coordination tests. An increased cerebrospinal fluid (CSF) protein level was detected during a cerebrospinal fluid investigation. The following findings were noted in the Nerve Conduction Velocity (NCV) study: (A) Motor Conduction - Median nerve: Compound Motor Action Potential (CMAP) amplitude (2.9 mV), Motor Conduction Velocity (MCV) (42 m/s); Ulnar nerve: CMAP (3.8 mV), MCV (37.2 m/s); Common Peroneal: CMAP (0.3mV), MCV (42.6 m/s); and Tibial Nerve: CMAP (05 mV), MCV (36.9 m/s); and (B) Sensory Conduction - Median nerve: Sensory Nerve Action Potential (SNAP) amplitude (20.1 mV), Sensory Conduction Velocity (SCV) (59.4 m/s); Ulnar nerve: SNAP (12.5 mV), SCV (57.2); and Sural nerve: SNAP (15 mV), SCV (47.1). F waves were normal in latency and persistence in Median, Ulnar and Tibial nerves. The NCV study was suggestive of demyelinating polyneuropathy. MRI revealed a small late subacute non-hemorrhagic infarct in the right parietal cortical and subcortical region.

**Table 1:** Demonstrates the variation in reflexes grading between pre- and post-treatment B/L = Bilateral Grading of reflexes: 0 = Absent, 1+ = Present but depressed, 2+ = Brisk response; normal, 3+ = Very brisk response, 4+ = Clonus; always abnormal

Types of reflexes	Pre-physiotherapy B/L	Post-physiotherapy B/L
Superficial Reflexes		
Corneal	1+	2+
Planter	1+	2+
Abdominal	1+	2+
Deep Reflexes		
Biceps	0	1+
Triceps	0	1+
Supinator	0	1+
Knee	0	1+
Ankle	0	1+

Timeline of the current episode

The onset of symptoms began on November 21, 2021. The patient was admitted to the hospital on December 1, 2021, and the necessary investigations were done on the same day; physiotherapy intervention began on December 2, 2021, and the patient was discharged from the hospital unit on 17 January 2022.

Therapeutic intervention

**Figure 3:**
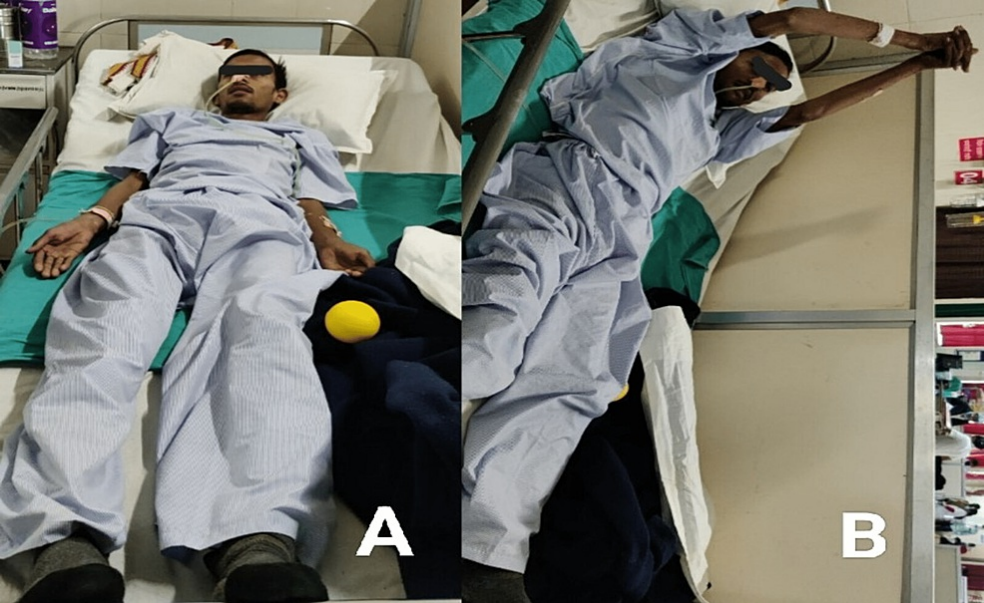
The status of the patient on the first day of physical therapy (A): Suggests the patient was bedridden and unable to initiate activities of daily living. (B): Patient was performing active exercises of the upper extremity.

Early rehabilitation was started following hospitalization (Figure 3). The term "rehabilitation" encompasses a wide range of services. indeed, before the development of medicines aimed at stabilizing the suspected immune system defects that underpin GBS, physical therapy was the only option. Physical therapy plays a crucial role in the treatment of this illness. The objective of therapy is to optimize neuromuscular functional recovery and eventually return to normal life as it was before GBS. The Frenkel exercises were included in the therapeutic plan because the patient had impaired coordination. These exercises are demonstrated in Table 2.

**Table 2:** Frenkel’s exercises while lying, sitting, and standing

Exercises while lying	Exercises while sitting	Exercises while standing
1) Flexion and extension of one leg by dragging the heel down in a linear fashion on the couch.	1) The heel of one leg slide to a spot indicated by a marking on the floor.	1) Weight is transmitted from one foot to the other during a stride standing.
2) Keeping knee flexed and feet on the couch, effortlessly abduct and adduct hip.	2) The heel of the contralateral side is placed at the designated location.	2) Step forward and backwards in a straight line.
3) By moving the entire leg on the table, abduct and adduct the leg with hips and knees extended.	3) Step up from a sitting position to a standing position with support.	3)Walk in a parallel bar.
4) Flex and lengthen the hip and knee while keeping the heel off the couch.	4) Stand and sit back with your knees together.	4) Place your foot on the marked point.
5) Flexion and extension of both legs at the same time, with the heel on the table.	5) In sitting performed hip abduction and adduction.	5) Turn around and walk again.
6) Extend one leg while flexing the other.	6) Place a hand on specified markers on the table.	6) walk in a different direction on command
7) Flexion and extension of one leg while abducting and adducting the other leg.	7) Placing your hand in the ring, attempt to reach and pick up the object.	7) Swing your arms forward and backwards while holding two sticks
8) The heel of the limb slides to the shin of the tibia on the opposite leg.	8) Bilateral pronation and supination of the hand.	8) Bouncing and catching a ball or tossing a ball

Phase one (0-2 weeks)

As the patient was bedridden, the following line of treatment was administered to prevent further complications, enhance strength, and maintain joint mobility. Frenkel's exercises were started in supine position. To avoid bed sores and deep vein thrombosis (DVTs), the patient was on air mattresses, and the weight of the leg could be supported occasionally by a cushion or foam pad beneath the calf to keep the heel off the mattress. Heel pads should have a right-angle form and a Velcro strap to keep them in place. To avoid deep vein thrombosis, ankle-toe motions were performed 15-20 times every 2-3 hours, and a compression bandage was recommended to prevent oedema. To avoid more complications, bed mobility activities were started. To optimize pulmonary functions, in order to alleviate dyspnea, the patient was given pursed-lip breathing exercises, deep breathing, and segmental breathing exercises for 10 repetitions three times a day, ensuring that all areas of the lungs were expanded. To maintain bronchial hygiene, the patient was given postural drainage three times a day. To allow for appropriate lung drainage, the patient lay face down on the bed, head hanging over the side. Manual claps and vibrations on the patient's chest in various regions were applied to dislodge collected mucus and assist the patient in coughing out the secretions. To retrain bladder and bowel functioning, the patient was instructed to tighten pelvic floor muscles by contracting glutes, hold the contraction for three seconds, and then release it for another three seconds. Repeat it several times throughout the day. Avoid flexing the muscles in your abdomen, thighs, and buttocks. Avoid holding your breath. Instead, take deep breaths throughout the workouts. Three times a day is a good rule of thumb. Aim for three sets of 10 repetitions every day. To preserve anatomical joint mobility, at least 10 repetitions of the passive movement were given three times a day. The patient preferred these procedures because of the tension that built up in his limbs. Multi-joint muscle groups were stretched completely. To re-educate the performance of functional activities, bed mobility activities such as rolling, supine to side-lying, and side-lying to prone, prone on hands, prone on the elbow, and sitting with support were initiated.

Phase two (2-4 weeks)

The patient was able to move a limb against gravity; however, tremors were observed due to weakness. The patient regained trunk control and was able to sit with minimal assistance. Frenkel’s were started and practised in a sitting posture. Exercises from phase one were carried over into phase two along with new ones. To enhance range of motion (ROM) and alleviate tightness, the hamstrings, quadriceps, gluteus maximus, tendo-achilles, biceps, and pectorals were all stretched. Stretching exercises were done three times during the day for ten repetitions of 15-30 seconds each. Active-assisted ROM movements were performed both bilateral to the upper and lower limb, three times each day for a minimum of 10 repetitions. To restore muscle strength, the upper and lower limb joints were both subjected to active movement with weight. Dynamic strengthening exercises were started bilateral to upper and lower limbs, three times a day with 15 repetitions each time, which were progressed to five times a day with 20 repetitions each time, once the patient gained a sufficient amount of strength. The bridging exercise was initiated, which increases hip extensor muscle strength and promotes trunk stability. Strengthening exercises were added which included isometric, isotonic, or isokinetic exercises with 10 repetitions of five-second hold, three times a day. To improve endurance, rather than increasing strength, low-resistance workouts were employed to develop stamina. Rather than a few repetitions against larger resistance, the patient completed several repetitions of activity against minimum resistance. Repetition with minimal weight improved muscular endurance as well as increased oxidative muscle capacity. This is referred to as aerobic training. To improve coordination activities, the coordination exercises were chosen to address the required movement capabilities of interest for the individual patient based on information collected from preliminary observation of functional activities. Following the completion of the nonequilibrium tests (finger-to-nose, finger-to-finger, finger-to-therapist finger, alternate heel to the shin, toe-to-examiners finger, etc.), the equilibrium tests are performed. The patient was experiencing trouble in performing equilibrium tests (standing in a normal posture, standing with feet together, etc). To regain fascial muscle strength, as the facial muscles were denervated, the galvanic current was given at 80 peaks/sec using a pen electrode for 30 repetitions of two sets.

Phase three (4-6 weeks)

The patient was capable of standing with minimum assistance and a wide base of support, his strength had increased significantly, and he was able to execute resistance movements. In phase three, the exercises from phases one and two were carried over, as well as new ones were introduced. To improve muscle strength and gait training, strengthening exercises were started with 10 repetitions three times a day with resistance using weight cuffs of the appropriate weight or physical resistance bands with the suitable colour code pattern. As the patient's strength will improve, he'll be able to lift larger weights for more repetitions. With the support of a walker, the patient was able to stand with minimum assistance, standing on the spot, marching on the spot, and standing with feet together. Walking with the help of a walker was first mastered, followed by walking with two crutches in a four-point gait pattern, then a two-point gait pattern, for six minutes three times a day, and lastly walking with the aid of a stick. To improve coordination activities, the patient could now readily and repeatedly execute basic non-equilibrium and equilibrium tests 2-3 times per day, with 10 repetitions of each test. To maintain fitness using aerobic exercises, low-intensity workouts and moderate-intensity workouts were done. Exercise modes like stationary bicycle, treadmill, and walking were adjusted to the patient's capacity and preference at first. To regain functional action of fascial muscles, for muscle reeducation, surged faradic with facial muscle actions were started with 30 repetitions and two sets per day. The patient was taught about facial proprioceptive neuromuscular facilitation.

Phase four (home-exercise program)

The patient was told to perform all or most of the exercises from phases two and three. To avoid difficulties and to provide aid as needed, the patient's family was advised to monitor the patient while exercising. The patient was advised to come to the rehabilitation centre in fifteen days for a follow-up appointment. Exercise progression was taught to the patient. Passive stretching may be used as an initial treatment, followed by low-resistance activities to increase endurance. In recovering GBS patients, fatigue is common, and strength and stamina should be gradually increased.

Follow-up and outcome of interventions

**Figure 4:**
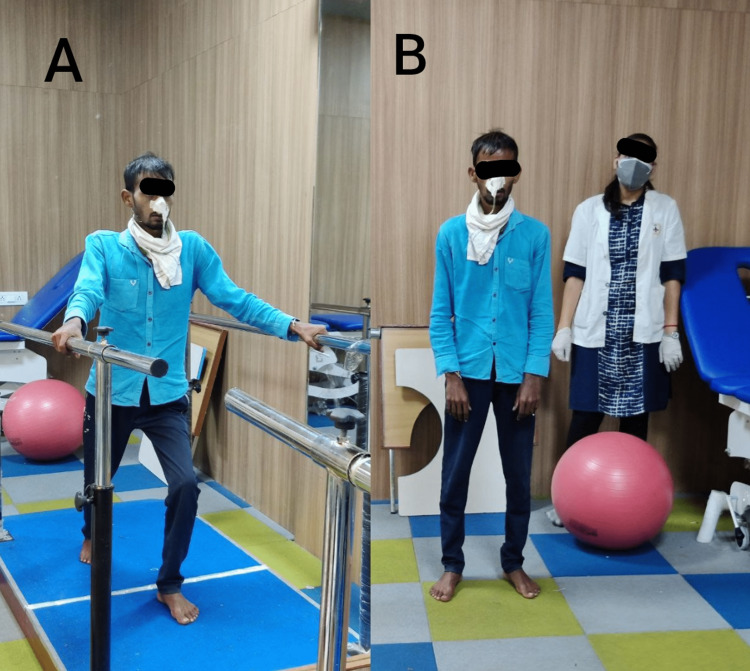
Patient status after six weeks of physical therapy (A): The patient was efficiently performing parallel bar activities. (B): After six weeks of the regimen, the patient was able to stand independently.

After six weeks of therapy, the patient came to the rehabilitation centre for further evaluation as shown in Figure 4.

The patient was taught advanced exercises after the assessment. Exercises on a floor mat were started once strength had been gained. These exercises are done with the therapist helping the movement at first, then against gravity, and lastly with weights as resistance. For example, a mat workout is hip abduction strengthening. The patient steadily elevates their upper leg while lying on their side, first with the therapist supporting a portion of the weight, then against gravity, and lastly against increasing resistance given by weights or springs. This aids in the development of strength of the hip abduction muscles. His treatment regimen includes thirty-minute sessions three times per week. The measurements taken were as follows: Manual Muscle Testing, pre- and post-treatment, was explained in Figure 1 and Figure 2. Hughes Functional Score was 2/6 pre-treatment and 4/6 post-treatment. Functional Independence Measure was 44/126 before treatment and 100/126 after treatment.

## Discussion

The goal of this case study was to demonstrate how physiotherapy rehabilitation has proven to have a positive impact on a patient with GBS. To the best of the authors' knowledge, there has been minimal research to guide physiotherapeutic approaches for patients with MFS specifically, hence standard rehabilitation strategies are based on what has been applied in the report. In this case report, the patient's recovery was further evidenced by an improvement in the primary outcome. GBS, MFS, and BBE are often monophasic, however, some patients endure recurrences after long periods of being asymptomatic. This study looked into the clinical characteristics of recurrent GBS, MFS, and BBE at a single institution. According to the findings of this study, sequelae occurred more often in individuals with MFS or BBE than in those with GBS [8]. A patient with MFS-GBS benefited from physiotherapy intervention in terms of functional outcomes. Patients who are unable to recover after six months might benefit from ongoing rehabilitation. Other therapeutic strategies, such as aquatic therapy, may have to be evaluated [9]. Prada et al. conducted a study on the value of intense and continuous rehabilitative treatment for GBS in 2019. The purpose of the study was to evaluate the impacts of the continuing significance of therapy combined with medicinal therapy on the disease's outcome [10]. Meythaler researched the therapeutic results of GBS patients. The patient's performance level improved, resulting in improving quality of life and a better functional outcome. As a result, this case study demonstrates that physiotherapy has a significant effect on improving strength, activities of daily living, and quality of life [11]. In a case study conducted by Bhagat and Brown in 2021, an atypical development of GBS with a positive anti-GD1b antibody presenting with acute facial diplegia, normal DTR, and delayed sensory ataxia were discovered [12]. This clinical case study illustrates the effect of physiotherapy interventions on encouraging functional restoration in people with MFS-GBS. It emphasizes the severity and persistence of symptoms, which can challenge a typical treatment approach, necessitating greater investigation into the impact of innovative interventions in supporting recovery.

## Conclusions

From the above findings, we concluded that a structured inpatient rehabilitation program, as well as a home exercise program, plays a crucial role in getting an optimal functional recovery in a patient suffering from MFS. As per the current condition of the patient, he is totally independent to perform all the activities of daily living and the therapist was able to differentiate between the body structure and function before and after the rehabilitation.
